# The Role of Capsid in the Early Steps of HIV-1 Infection: New Insights into the Core of the Matter

**DOI:** 10.3390/v13061161

**Published:** 2021-06-17

**Authors:** Nawal AlBurtamani, Alwin Paul, Ariberto Fassati

**Affiliations:** Division of Infection & Immunity, Institute of Immunity and Transplantation, University College London, London WC1E 6JF, UK; nawal.burtamani.17@ucl.ac.uk (N.A.); alwinpaul1324@gmail.com (A.P.)

**Keywords:** HIV-1, capsid, reverse transcription, nuclear entry, integration, drugs

## Abstract

In recent years, major advances in research and experimental approaches have significantly increased our knowledge on the role of the HIV-1 capsid in the virus life cycle, from reverse transcription to integration and gene expression. This makes the capsid protein a good pharmacological target to inhibit HIV-1 replication. This review covers our current understanding of the role of the viral capsid in the HIV-1 life cycle and its interaction with different host factors that enable reverse transcription, trafficking towards the nucleus, nuclear import and integration into host chromosomes. It also describes different promising small molecules, some of them in clinical trials, as potential targets for HIV-1 therapy.

## 1. Introduction

Human immunodeficiency virus type 1 (HIV-1) is a lentivirus within the family of Retroviridae, and it is the causative agent of the acquired immunodeficiency syndrome (AIDS). HIV-1 infects CD4+ cells, whether dividing or not, and CD4+ helper T cells represent its main target. HIV-1 replication starts by the viral fusion to the cell plasma membrane [1,2]. At this point, the capsid core, which encases the viral RNA, is released into the cytosol of the infected cells initiating the viral journey towards the nucleus, while the viral RNA genome is reverse transcribed into a double-stranded DNA molecule [3]. In its mature state, the HIV-1 capsid has a fullerene conical shape and is composed of ~1300 copies of CA p24 proteins arranged in hexamers and pentamers [4,5]. It contains the viral RNA genome and associated proteins, including nucleocapsid (NC), reverse transcriptase (RT), and integrase (IN) [6]. It is recognised that the HIV-1 capsid core plays crucial roles in nearly every stage of the early viral replication cycle. It promotes reverse transcription forming the so-called reverse transcription complex or RTC [7,8,9], it interacts with host cellular factors and participates in viral trafficking, uncoating, nuclear import and integration [1,7]. The viral capsid core can be targeted by cellular restriction factors such as TRIM5α and MX2 [10,11] and is stabilised by host factors (reviewed in [1]). The highly ordered nature of the HIV-1 capsid core and its integrity are important for viral fitness and infectivity. Thus, the HIV-1 capsid is also an important target for antiviral drug development. Here, we review the role played by the HIV-1 capsid in the early steps of the HIV-1 life cycle, from reverse transcription, to intracellular trafficking, to nuclear import and integration. Lastly, we briefly describe drugs and small molecules that target the capsid and perturb these events.

## 2. The Capsid Structure Reveals Several Binding Pockets

The capsid protein (CA p24 in HIV-1) is generated by proteolytic cleavage of group-specific antigen protein (Gag) and Gag-pol polyproteins, which are encoded by the *gag* and *gag-pol* genes of the integrated provirus (reviewed in [4]).

**Figure 1:**
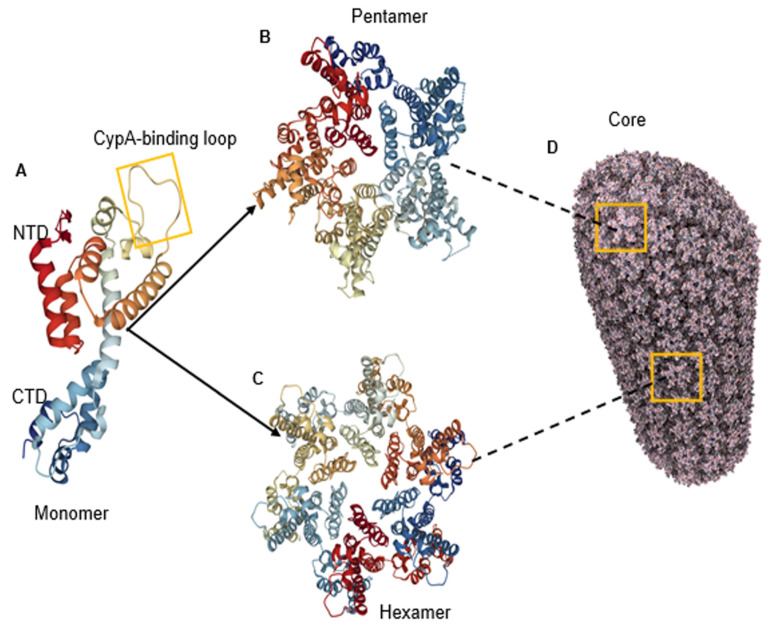
Structure of HIV-1 capsid. (**A**) The structure of the CA monomer showing the N-terminal domain (NTD), the C-terminal domain and the CypA-binding loop (highlighted) (PDB 4XFY) [20]. (**B**) The structure of pentameric HIV-1 CA (PDB 3P05) [21]. (**C**) The structure of hexameric HIV-1 CA (PDB 4XFY) [20]. (**D**) The hexameric and pentameric subunits assemble into a fullerene conical capsid core (PDB 3J3Y). In this model, the core is composed of 186 hexamers and 12 pentamers [22].

In order for HIV-1 to build its mature capsid, two stages are required. In the first stage, Gag is translated as a polyprotein and oligomerises into a lattice. This requires around 2000–5000 copies of Gag polyprotein to assemble into incomplete spherical capsid cores [12,13], thus forming the immature non-infectious virion that buds out from the cell. In the second stage, which occurs immediately after virus budding, the viral enzyme protease (PR) cleaves the Gag in multiple places to yield the mature proteins matrix (MA); capsid (CA), nucleocapsid (NC) and p6 domains; and two spacer peptides, SP1 and SP2 (reviewed in [14]). This produces a dramatic rearrangement of the capsid core in a process called viral maturation. Maturation results in the assembly of approximately 200–250 capsid hexamers and 12 pentamers from 2500 CA monomers to form the mature cone or fullerene-shaped capsid core (Figure 1A–D) [15]. The 12 pentamers are distributed at the capsid ends, 5 at the narrow and 7 at the wide end, facilitating the curvature of the lattice and its enclosure [15,16]. The hexamer and pentamer building blocks are called capsomers and are composed of six CA monomers for the hexamer or five for the pentamer (Figure 1A–C), each one composed of two alpha-helical domains, namely, an N-terminal domain (NTD) (helices 1–7) and a C-terminal domain (CTD) (helices 8–11), which are separated by a short flexible linker (Figure 1A) [4]. The NTD also contains a cyclophilin A (CypA)-binding loop (Figure 1A) [17,18,19].

Recent crystallographic data using native CA demonstrated that the hexameric lattice is more flexible than originally thought due to water-mediated hydrogen bonds at side chains of certain conserved residues important for CA–CA interactions [20]. Near-atomic reconstruction of the HIV-1 capsid core by cryo-EM also revealed that the NTD–CTD linker domains are flexible, allowing some degree of asymmetry of the capsid hexamer, which can curve at an angle of about 20°, thus contributing to the overall curvature of the capsid core itself [16,23]. In agreement with this notion, molecular dynamics simulations predicted that the capsid core oscillates, suggesting that structural flexibility might allow the core potentially to adopt different conformations in response to allosteric cues, such as co-factor binding [24] or reverse transcription [25]. 

**Figure 2:**
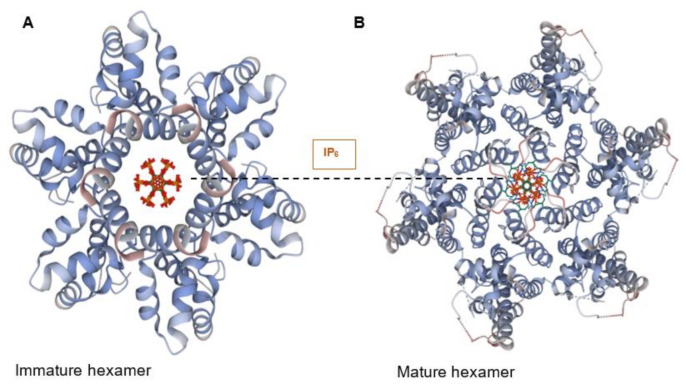
IP_6_ binds to CA hexamers. (**A**) IP_6_ bound to the central channel of the immature capsid hexamer (PDB 6BHR) [29]. (**B**) IP_6_ bound to the Arg18 ring in the central channel of the mature CA hexamer (PDB 6ES8) [28].

At the centre of each CA hexamer is a ring of six arginine residues (residue R18). The ring is formed by β-hairpins of the CA NTD oriented to form a “channel” at the sixfold axis of the capsid hexamer [26]. The centre of this ring is positively charged due to the presence of arginine (Arg) residues and can bind both dNTPs and rNTPs [26]. Importantly, the ring also binds inositol hexa-phosphate (IP_6_), a small and abundant intracellular polyanion [27] that promotes assembly of the immature capsid core during budding and also promotes correct CA assembly in the mature core, even stabilising the incoming capsid core during the early stages of infection (Figure 2A,B) [28,29]. 

In addition to the IP_6_-binding channel, the capsid hexamer contains several regions that are recognised by cellular factors. These include the exposed CypA-binding loop at each NTD [30,31] and a cleft formed at the interface between the NTD of one CA monomer and the CTD of another lined by the side chains of Pro34, Ile37, Pro38, Asn53, Leu56, Asn57, Val59, Val142 and Gln176 [32], which binds host factors nucleoporin 153 (Nup153) and cleavage and polyadenylation specificity factor 6 (CPSF6) (see below). These advances in our understanding of the HIV-1 capsid core structure have illuminated several of its functions from reverse transcription to nuclear import, as described below.

## 3. CA and Reverse Transcription

Reverse transcription is an obligatory step and one of the defining characteristics of retrovirus replication, in addition to integration [3]. Reverse transcription takes place in a large ribonucleoprotein structure called the reverse transcription complex (RTC) that, besides the reverse transcriptase (RT) enzyme, consists of the RNA genome coated with nucleocapsid proteins (NC) and other components, such as CA and viral protein R (Vpr) molecules [33,34]. The nature of the RTC has been hotly debated for many years, but more recently, a consensus has been reached that RTCs are in fact progressively uncoating capsid cores [35]. The first studies characterising the intracellular RTC in acutely infected cells made use of sucrose sedimentation velocity and density gradient centrifugation. RTCs showed a high density, consistent with that of a ribonucleoprotein complex, and their sedimentation velocity decreased from 560S at 1 h to 220S and 80S at 16 h post-infection, suggesting that they were becoming smaller [34,36]. After density centrifugation, viral DNA, reverse transcriptase, integrase, the accessory protein Vpr and histones were found to be associated with the RTC, but CA was not detected [34,36]. In contrast, in the same gradient centrifugation assays, substantial amounts of capsid were found to be associated with RTCs from Moloney leukaemia virus [37]. When provided with exogenous dNTPs, the sucrose fractions containing HIV-1 RTCs could synthesise (+) strand viral DNA (a late RT product), albeit at low efficiency [34].

Subsequent studies based on imaging detected CA associated with a significant proportion of RTCs during reverse transcription [8,38,39] and even intact cores in proximity of the nuclear envelope [8,39,40]. Genetic approaches showed that mutant virus with capsid cores that were intrinsically less stable than wild-type cores synthesised viral DNA inefficiently, indicating that integrity of the core promoted reverse transcription [41]. However, the same genetic approaches showed that mutant viruses with intrinsically hyper-stable capsid cores were also defective in reverse transcription, suggesting that some degree of capsid loss was necessary [41]. Studies that employed indirect functional readouts to measure the integrity of the capsid core shortly after infection, based on the core-destabilising effects of the capsid-binding TRIM5α protein, or the drug cyclosporin A, suggested that CA was progressively lost as reverse transcription progressed [42,43,44]. This phenotype was attributed to the increasing mechanical stress exerted on the core itself by the conversion of compacted RNA into less compacted single stranded DNA and then a more rigid double-stranded DNA molecule as reverse transcription proceeds. It has been problematic to reconcile these different lines of evidence, not least because the assumption was that the stability of the capsid core would be an “all or nothing” phenomenon whereby even loss of small amounts of CA would trigger a catastrophic collapse of the entire structure [45,46]. 

The discovery that IP_6_ is incorporated into capsid cores and stabilises them represented a significant step forward [28]. Biochemical experiments showed that isolated viral cores carry out reverse transcription more efficiently in the presence of IP_6_ and disassemble with slower kinetics than cores in its absence [28,47]. These results suggested that an intact capsid core facilitates reverse transcription and also explained why CA was not found associated with HIV-1 RTCs using early biochemical assays: IP_6_ was not present in the buffers used for density gradient centrifugation, which therefore revealed the intrinsic instability of the HIV-1 capsid core and the greater intrinsic stability of the Moloney murine leukaemia virus core [34,37]. However, recent cryoEM evidence showed that reverse transcription carried out in vitro in the presence of IP_6_ generates different capsid core morphologies, ranging from intact to 50% disassembled [48]. Many capsid cores had lost patches of hexamers and presented DNA loops extruding from the core itself, which demonstrated that core disassembly needs not happen in an “all-or-none” fashion but can in fact be partial and progressive [48]. Even a modest loss of CA from the capsid core would allow the influx of dNTPs and promote reverse transcription, although the central channel has also been suggested to facilitate the influx of dNTPs [26], and indeed both mechanisms may be operating at different stages of reverse transcription [49]. It is therefore likely that the observed changes in sedimentation velocity of the RTCs at different times post-infection [34] reflect the different capsid core morphologies brought forward by the conversion of the viral genome from RNA into a double-stranded DNA. This notion is supported by recent atomic force microscopy analysis of the capsid core in the presence of IP_6_, showing step-wise, discrete core deformations that coincide with the three steps of reverse transcription, with progressive loss of core integrity [25]. 

It is not clear if such RTCs containing partially disassembled cores are sufficiently stable to proceed to nuclear import and integration [9,50]. Exposed viral nucleic acids may be rapidly degraded by intracellular nucleases such as TREX [51], which would then eliminate unstable capsid cores in a kind of intra-cytoplasmatic “purifying” step. Expression levels of TREX in different cell types may therefore be critical in determining the fate of the RTCs and explain why in cell lines, with presumably low levels of TREX, significant amounts of “uncoated” double-stranded viral DNA can be detected in the cytosol for hours after infection [34,52,53]. 

Remarkably, HIV-1 evades innate immune sensing in macrophages [54,55,56] and in CD4 T-cells [57]. Instability of the capsid core during reverse transcription was shown to trigger sensing of the viral DNA by cGAS in macrophages and monocytic-like THP-1 cells [58]. The triphosphohydrolase SAMHD1 has also been shown to reduce the interferon (IFN)-type 1 response triggered by the cGAS-STING pathway, presumably because fewer viral DNA molecules are synthesised when dNTPs concentration is lowered by SAMHD1 [59]. However, capsid destabilisation did not seem to trigger cGAS sensing in CD4 T-cells [57]. Moreover, it is worth noting that the viral DNA in purified RTCs seems quite resistant to nuclease digestion [34,60], and fully reverse-transcribed, integration-competent “pre-integration complexes” can be extracted from the cytoplasm of acutely infected cells [61,62], which suggests that sensing of viral nucleic acids may be suppressed by more than one mechanism.

Recent evidence supports the notion that there are multiple mechanisms for HIV-1 immune evasion. Vpr has been shown to block nuclear import of IRF3 and NF-kB, which are key transcription factors that induce multiple interferon stimulated genes [63]. N6-methyladenosine modification, which is found on the HIV-1 RNA, has been reported to prevent its sensing by RIG-I in the cytosol of myeloid cells [64]. Furthermore, recent evidence based on imaging showed that reverse transcription can also be completed inside the nucleus [65,66], which agrees with the observation that significant amounts of viral RNA can be detected in the nucleus shortly after infection [67]. The rapid transport of the capsid core into the nucleus, before reverse transcription is completed, would reduce the chances of the viral DNA being detected by cGAS in the cytosol [68,69]. 

## 4. CA and Cytoplasmic Trafficking

After fusion and entry into the host cell cytoplasm, HIV-1 virus must traffic towards the nucleus in order to integrate its genome into host chromatin. HIV-1 exploits microtubules and microfilaments to reach the nucleus by the so called retrograde movement [38,70], and CA has been identified as a key player in facilitating this process (reviewed in [8]). The cytoskeleton comprises three types of networks: microtubules (MT), intermediate filaments and actin filaments, also called microfilaments. MTs are hollow tubes, and their structure comprises 13–15 protofilaments of α-/β-tubulin heteropolymers with their plus ends oriented towards the cellular periphery and minus ends attached to the centrosome or microtubule organising complex (MTOC) [71]. Two types of motor proteins appear to mediate the HIV-1 MT transport movement, dynein and kinesin. Dynein mediates the retrograde movement towards MT minus end and is composed of heavy, intermediate and light chains [72,73,74], whereas kinesin mediates the anterograde movement towards MT plus end [72,73,74]. McDonald et al. first demonstrated that HIV-1 moves along MTs in a dynein dependent manner [38]. Since then, several studies have identified a number of cellular proteins that interact with HIV-1 CA and promote retrograde movement. Earlier studies showed that MT stabilisation is induced by plus-end tracking proteins (+TIPs) that are recruited by protein EB1 [75,76,77], and this stabilisation is critical for HIV-1 trafficking towards the nucleus. Implementing a yeast two-hybrid screen, Fernandez and colleagues [77] identified two microtubule-associated proteins, namely MAP1A and MAP1S, that interact with HIV-1 CA early in infection. This leads to the formation of stable microtubules and to tethering the incoming viral particle to the MT, thus promoting efficient cytoplasmic trafficking [78]. This interaction is lost upon MAP1 depletion, resulting in impaired retrograde trafficking [78]. The Naghavi lab investigated how +TIPs bind to the HIV-1 core, thus inducing MT stabilisation and promoting HIV-1 infection. They found that EB1-associated +TIPs involved in MT stabilisation do interact with the HIV-1 capsid in early HIV-1 infection. Firstly, they reported that Kif4, an EB1 binding protein, is targeted by the HIV-1 matrix to recruit EB1 to the +TIP and induce MT stabilisation. They also showed that depletion of EB1 prevented MT stability and suppressed early infection [79]. Secondly, they identified other +TIPs that are recruited by HIV-1 CA and stabilise MTs; the diaphanous-related formins (DRFs) Dia 1 and Dia 2 [80] and cytoplasmic linker-associated protein 2 (CLASP2) [81]. Dia 1 and Dia 2 were shown to induce MT stabilisation, promote HIV-1 trafficking and coordinate capsid core uncoating [80]. Their recent study identified CLASP2, a +TIP that enables filament stabilisation, to promote HIV-1 trafficking to the nucleus through its C-terminal domain binding to viral capsid [81]. Two independent studies reported that protein bicaudal D2 (BICD2), a dynein adapter protein that activates dynein-mediated transport, is utilised by HIV-1 for cytoplasmic trafficking [82,83]. They found that BICD2, via its CC3 domain, binds to CA and facilitates its trafficking to the nucleus. Moreover, cell depletion of BICD2 resulted in reduced HIV-1 retrograde movement [83] and innate immune sensing [82]. 

Early live cell imaging observed a bi-directional movement, retrograde and anterograde, of HIV-1 on microtubules [38,70]. This led to the identification of fasciculation and elongation factor zeta 1 (FEZ1), which is a kinesin-1 adaptor protein [84]. FEZ1 was shown to bind HIV-1 capsid hexamers directly and link these to kinesin motors [84,85]. Further to this, FEZ1 was shown to compete with IP_6_ and dNTPs for capsid interaction [85], and its depletion impeded early HIV-1 infection and trafficking to the nucleus [84]. The interaction between FEZ1 and HIV-1 capsid promotes net retrograde transport.

## 5. CA and Nuclear Import

HIV-1 has a remarkable ability to enter the nucleus of non-dividing cells for integration into host chromatin. This is in contrast to oncoretroviruses, such a Moloney murine leukaemia virus (MoMLV), which require breakdown of the nuclear envelope to access chromatin [86]. Although initial efforts to understand HIV-1 nuclear import were focused on the karyophilic properties of matrix, integrase, Vpr, tRNAs incorporated into viral particles and the central DNA flap [87,88,89], it later became apparent that CA was a key determinant. The importance of CA was first shown genetically when chimeric viruses were generated by replacing the HIV-1 capsid with the capsid of MoMLV [37,90,91]. Remarkably, a chimeric HIV-1 with MoMLV capsid could no longer infect non-dividing cells, which meant that CA was the determinant of this phenotype [37,90,91]. Further analyses supported the role of CA in HIV-1 nuclear import and mapped some key residues involved in the process [91,92].

The nuclear pore complex regulates passage of molecules in and out of the nucleus, and it is composed of a central scaffold with eightfold rotational symmetry around its central axis, which is embedded in the nuclear membrane. Anchored to the central scaffold are, on opposite sides, eight cytoplasmic filaments and the nuclear basket. A central channel occupies the middle of the nuclear pore complex and tightly regulates passage of molecules [93]. The entire nuclear pore complex is composed of approximately 30 nucleoporins (Nups), each present in many copies and assembled in subcomplexes in a modular fashion [94]. Genome-wide siRNA screenings revealed that several Nups that contain phenylalanine-glycine (FG) repeats, most notably Nup358 and Nup153, facilitate HIV-1 nuclear entry [95,96]. Nup358 (also called RanBP2) forms long filaments that protrude from the cytoplasmic ring of the nuclear pore complex, whereas Nup153 forms a basket at the nuclear side of the nuclear pore complex [93]. These findings were supported by studies employing stable depletion of Nups and imaging to show that Nup358 promoted docking of the HIV-1 RTC to the nuclear pore complex, whereas Nup153 stimulated actual translocation through the nuclear pore [97]. Nup124, also forming filaments protruding from the cytoplasmic NPC ring, did not appear to take part in HIV-1 nuclear import directly but inhibited HIV-1 infection indirectly by reducing mRNA export [97]. 

The connection between Nup358, Nup153 and CA soon became apparent. Nup358 contains a C-terminus CypA homology domain, which was found to interact with CA at low affinity and induce isomerisation of residue P90 [98,99]. This interaction was shown to be important for mediating RTC nuclear docking to the nuclear pore [97,100], although CA may also interact with Nup358 in a Cyp-A independent fashion [101]. Because there are eight Nup358 molecules protruding from each NPC, the low-affinity interaction with CA is strengthened by greater avidity, allowing enough time for the RTC further to engage with additional NPC components. Nup153 was shown to bind to HIV-1 capsid cores in vitro via its C-terminal FG motifs [102,103]. Notably, Nup153 binds to the cleft formed at the interface between helices 3–5 of the NTD of one CA monomer and the CTD of another (see above) [32,104], which explains why Nup153 binds poorly to monomeric CA but more strongly to hexameric CA [32]. 

Further genome wide siRNA screening and a subsequent yeast two-hybrid screening identified transportin 3 (TNPO3) as a host co-factor for HIV-1 infection [105,106]. TNPO3 is a β-karyopherin that, under normal physiological conditions, is involved in facilitating the nuclear entry of SR proteins/splicing factors [107]. Because TNPO3 is a karyopherin, it was initially assumed to have a direct role in nuclear import of the HIV-1 RTC. The phenotype was mapped to CA [108] and integrase [106,109,110], but subsequent studies presented a mixed picture, with some reports showing that TNPO3 indeed stimulated viral nuclear import [107,111,112,113] and other reports showing that TNPO3 was more important for HIV-1 integration [114,115,116,117,118]. Conclusive proof for the role of TNPO3 in HIV-1 integration was provided by studies using peripheral blood mononuclear cells (PBMCs) from limb girdle muscular dystrophy 1F patients [119]. These patients have a mutation in the *TNPO3* gene that makes them resistant to HIV-1 infection. In these patients, HIV-1 integration was drastically reduced, but nuclear import was not [119]. The relatively modest inhibition of nuclear import observed in the absence of TNPO3 maps mainly to CA and can be explained by an indirect mechanism. Indeed, TNPO3 binds to host factor CPSF6 and transports it into the nucleus [120]. Lower TNPO3 levels result in an aberrant accumulation of CPSF6 in the cytoplasm, which binds to the capsid core [111,120,121]. CPSF6 and Nup153 compete for binding to the same cleft in the hexameric CA [32]; hence, the premature engagement of the capsid core with CPSF6 in the cytoplasm is likely to inhibit Nup153 binding at the nuclear pore complex, reducing the nuclear import efficiency of the RTC. TNPO3 binding to integrase has also been shown to promote HIV-1 nuclear import [106,109,110].

Remarkably, it appears that the interactions between CA, Nup358 and Nup153 are sufficient but not necessary for HIV-1 nuclear import. HIV-1 bearing capsid mutations N74D, N77V and P90A, which do not interact with these host factors, is still able to access the nucleus and integrate, albeit with a different integration site selection profile [99,114,122,123,124]. This indicates that there is a substantial degree of flexibility in the HIV-1 nuclear import mechanisms [123], which may be in part attributed to the heterogeneity of the size and perhaps even composition of nuclear pore complexes [94,125]. Currently, it is not clear if the CA mutants must still be part of the RTC but engage with alternative host factors, or if they are shed and nuclear import is mediated by other viral and cellular components. Not enough attention has been dedicated to these alternative nuclear import mechanisms, which may become clinically relevant, because mutant N74D emerges following treatment with lenacapavir, a new and highly potent capsid-targeting antiretroviral (Sorana Segal-Maurer, CROI 2021, abstract 128). 

The nuclear pore complex is characterised by a central channel composed of disordered and filamentous FG-rich nucleoporins, which together form the selectivity barrier. This barrier is highly hydrophobic and filters out molecules larger than about 5 nm, which need to bind to specialised nuclear transport receptors (NTRs, also called importins or karyopherins) to be “chaperoned” across the nuclear channel [93]. The maximum functional size of the central channel was estimated to be about 40 nm [126]. Because the diameter of the HIV-1 capsid core at the wide end is about 60 nm, it was assumed that the core needed to either uncoat or rearrange structurally to be accommodated through the central channel. Loss of significant amounts of CA from the core was indeed observed before the completion of nuclear import [100,127], and transportin-1 (TNPO-1) has been implicated in this uncoating step by directly binding to CA and destabilising the capsid core structure [128]. However, this assumption has been recently questioned by several independent studies showing that intact or near-intact cores can be observed inside nuclear pores and, in some cases, even inside the nucleus [52,66,129]. It has also been reported that Nup153 may stabilise the capsid core or its remnants at the nuclear basket [130]. Most intranuclear viral capsid cores detected were, however, more spherical and smaller than the typical conical-shaped core, suggesting some kind of structural rearrangement [52,129]. These interesting observations indicated that the functional diameter of the central channel may be greater than 60 nm. Nuclear pores are to some degree subject to mechanosensing. In particular, it has been proposed that nuclear import may increase when nuclei are subjected to mechanical force, flattened or spread out on a rigid surface, presumably because the central channel becomes more permeable [131]. The Nup153 basket can dilate in response to cytoskeletal-induced tension through its interactions with pore protein SUN1 and cytoskeletal components, such as talins, LINC and nespins, although no size changes have yet been detected in the central channel itself [132].

Even if the central channel will prove to be larger than 60 nm in diameter, the question of how the large capsid core can traverse the tight hydrophobic transport barrier remains unanswered. Atomic force microscopy analyses on native NPC in physiological buffer indicated that the FG-Nups are confined in the central channel at a very high density and form a dynamic gel through their intermolecular interactions, which may be gradually and locally displaced to make room for very large cargos [133,134]. In this scenario, intermolecular interactions between the FG-Nups would be weakened and replaced by FG-Nups/viral core interactions, inducing a partial and localised collapse of the FG-Nups towards the wall of the central channel [133,135]. This “bi-stable” behaviour of the Nups inside the channel may explain how the viral core manages to go across the nuclear pore.

## 6. CA and Integration 

The link between HIV-1 integration and CA was first suspected based on the observation that pre-integration complexes (PICs) obtained from viruses with extra-stable capsids were incompetent for in vitro integration [136]; genetic evidence also pointed to a role of CA at a step post nuclear import [91]. The discovery that antibiotic coumermycin-A1 targeted CA and inhibited HIV-1 integration further supported this link [137]. Remarkably, passaging HIV-1 in the presence of coumermycin-A1 selected an escape variant with the A105S mutation in CA, which integrated at normal levels in the presence of the drug [137]. Coumermycin-A1 binds to hexameric CA, similar to another small molecule with antiretroviral activity developed by Pfizer called PF-74 [130,138]. These pharmacological findings connecting CA to integration have been recently corroborated by the development of second-generation CA inhibitors, such as GS-6207 (see also below), which potently block HIV-1 replication [139]. Molecular analysis of the steps of the HIV-1 life cycle affected by GS-6207 revealed a modest block to nuclear import but a dramatic block to integration [139], reminiscent of the defects observed in human cells that have mutations in TNPO3 [119].

It had been difficult to make sense of the early reports linking CA to integration, because it was widely assumed that CA did not enter the nucleus. However, in 2011, Zhou et al., by using cell fractionation as well as immunostaining of fixed cells, reported that some CA could in fact enter the nucleus, where it accumulated in greater amounts over time post-infection [118]. Zhou et al. also noted that the N74D mutant CA did not accumulate inside the nucleus but remained bound to the nuclear envelope or the periphery of the nucleus [118]. This study found a link between TNPO3, CA nuclear accumulation and HIV-1 integration and proposed that efficient integration required a “nuclear uncoating step” [118]. Initially greeted with scepticism, the observation that CA was present in the nucleus was later confirmed and expanded to show that nuclear CA was actually associated with the viral genome and even with a largely intact viral capsid core [100,140,141,142,143]. The N74D capsid mutant was consistently detected at the periphery of the nucleus using different experimental approaches [141,144,145]. Furthermore, the concept of a nuclear uncoating step has also received support based on EM and fluorescent imaging [52,66,129]. Taken together, the available evidence strongly suggests that proper uncoating in the nucleus is required for efficient integration. Whether the nuclear uncoating step is mediated by TNPO3, by host cell factors or by the completion of reverse transcription is an issue awaiting further research. 

**Figure 3:**
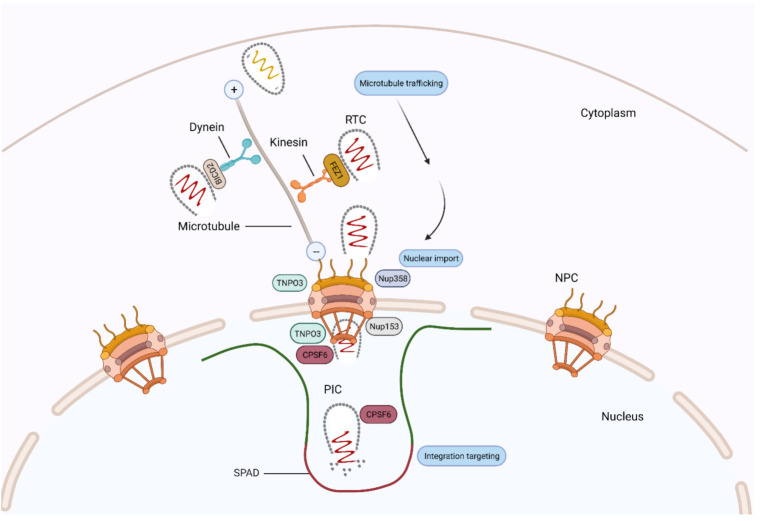
A model showing HIV-1 CA-interacting host factors during microtubule trafficking, nuclear import and integration targeting. Dynein adaptor protein BICD2 and kinesin-1 adaptor protein FEZ1 bind to HIV-1 CA and facilitate the HIV-1 reverse transcription complex (RTC)/pre-integration complex (PIC) trafficking towards the nuclear membrane. At the nuclear pore complex (NPC), Nup358 interacts with the CA protein and mediates the docking of RTC/PIC. Then, Nup153 interacts with the CA protein and mediates PIC translocation through the NPC. CPSF6 then binds to and directs CA to active regions of euchromatin, proximal to nuclear speckles and speckle-associated domains (SPADs). TNPO3 also facilitate virus integration, possibly by promoting nuclear core uncoating.

In addition to integration per se, CA has been shown to influence HIV-1 integration targeting. Since the publication of the landmark study by Schroeder et al. [146], it has been known that HIV-1 preferentially integrates into gene-dense regions and within highly expressed genes (reviewed in [88]). Integration targeting was shown to be dependent on the interaction between viral integrase and host factor LEDGF/p75 [88]; however, it later became apparent that depletion of host co-factors known to bind CA, such as Nup358, also changed the integration preference [114]. Furthermore, HIV-1 bearing certain CA mutants, including N74D, integrated in less gene-dense regions compared to the wild-type virus [99]. Host cell factor CPSF6 was noted to be the missing link between CA and integration targeting [147], and this link has been shown to be conserved among different primate lentiviruses, distinguishing these from non-primate lentiviruses [148]. CPSF6 is a nuclear protein that functions in processing mRNA for polyadenylation as a component of the mammalian cleavage factor 1 (CFIm). Three protein domains constitute the structure of CPSF6: an N-terminal RNA recognition motif [149]; a central proline-rich domain, which confers binding to CA [150]; and a C-terminal arginine/serine-rich domain that facilitates binding to TNPO3 [120]. As described above, CPSF6 and Nup153 recognise the same cleft in the hexameric capsid lattice, and certain CA mutations, such as N74D, abrogate this interaction [151]. By binding to capsid, CPSF6 directs intranuclear localisation of the virus to active regions of euchromatin, proximal to nuclear speckles and speckle-associated domains (SPADs) [69,148], which contain gene-dense and active chromosomal compartments [152,153]. Recent studies have shown that in the absence of this interaction, for example, by depleting CPSF6 or using mutant viruses that do not bind CPSF6 (e.g., in the N74D mutant), pre-integration complexes (PICs) mislocalise to the nuclear periphery ([144,145], mapping integrations to transcriptionally inactive regions of heterochromatin known as lamina associated domains (LADs) [154] (Figure 3).

Clearly, CPSF6 contributes to HIV-1 integration targeting to gene-dense and active chromatin regions, such as nuclear speckles; however, the functional significance of such a targeting has been rather difficult to prove, not least because HIV-1 infection does not seem to be greatly affected by either depletion of CPSF6 or by mutations in CA that abrogate binding to it [147]. The relevance of CA-directed integration targeting became more apparent upon perturbation of cellular gene expression. By performing RNAseq in Jurkat CD4-T cells, Zhyvoloup et al. [124] showed that the cardiac glycoside drug digoxin inhibited a network of genes important for glucose metabolism and T-cell activation. Digoxin was also found to repress HIV-1 gene expression. Remarkably, however, wild-type HIV-1 was more susceptible to digoxin than the N74D capsid mutant virus was, and this phenotype was clearly determined by the different integration site preference of the two viruses [124]. Further integration site analysis guided by this phenotype revealed that the wild-type virus integrated into genes important for glucose metabolism and T cell activation (the same genes whose transcription was affected by digoxin) much more frequently than the mutant N74D virus did [124]. This integration pattern made wild-type HIV-1 more susceptible to changes in activity of these genes when compared to the N74D virus, which integrated in different places. Therefore, the relevance of HIV-1 integration targeting may become apparent when CD4 T-cells undergo physiological changes that perturb their overall transcriptional program, for example, from quiescent to activated and vice versa, or from naïve to effector memory [155,156].

## 7. CA and Antiretroviral Drugs

CA is involved in every step of the early HIV-1 replication cycle, and it has therefore become a key target for drug development. The quest for antiretroviral drugs that target capsid has been rewarded by the recent clinical trial results for lenacapavir, a potent and long-acting antiretroviral that promises to be a game changer in HIV-1/AIDS therapy. In addition to lenacapavir, there are several other small molecules that show promise as described below (Table 1).

### 7.1. BVM

Bevirimat (BVM), formerly PA-457, was identified in 1994, through a screen for anti-HIV-1 compounds. This identified a betulinic acid derived compound, which was since chemically modified into BVM [157]. The primary mechanism by which BVM is suggested to act is late in the replication cycle by blocking the proteolytic cleavage of CA from its precursor, CA-SP1. This prevents maturation and triggers a morphological defect in virions produced by BVM-mediated replication [158,159,160]. Though putative, the binding site is suggested to be inside the six-helix bundle of the CA-SP1 region, therefore stabilising the complex and blocking PR-mediated cleavage [161,162,163]. This is also indicative that BVM binds to immature HIV-1 particles, as the binding site is absent following cleavage of CA in mature particles [164]. There is also evidence to suggest that BVM interacts with a region of the CA known as the major homology region (MHR), which may be involved in the mechanism of BVM inhibition. However, BVM did not progress in clinical trials due to the presence of pre-existing resistant HIV strains with SP1 polymorphisms that reduced BVM efficacy in a number of patients [160,165].

### 7.2. CAP-1

CAP-1 was first described in 2003, from screening of chemical libraries for small molecules that bound to the CA-NTD. EM analysis demonstrated that CAP-1 binding results in increased size heterogeneity in HIV-1 particles, though there is no significant quantitative change in viral production. This aberrant morphology causes a disruption in the CA–CA interactions needed for capsid assembly and maturation and parallels to that of previously studied CA mutants [166]. CAP-1 binds to the CA-NTD at the vertex of a 5-helix bundle—helices 1,2,3,4,7—in a deep hydrophobic pocket that is formed by the protuberance of the Phe32 side chain from a buried to exposed position [167]. Further studies have shown that CAP-1 also causes a significant conformational change at the binding site due to displacement of aromatic side chains, specifically F32, H62 and Y145, which contribute to a polar network that stabilises a loop formed by helices 3 and 4. Overall, this suggests that CAP-1 induced-fit binding and subsequent change to local geometry also inhibit capsid assembly by disrupting the intermolecular NTD–CTD interactions required for the formation of the CA hexamer [167,168]. 

### 7.3. Peptide Inhibitors/CA1 and NYAD-1

CA1 is a 12-mer-alpha-helical peptide that was discovered in a phage display screen in 2005 [169]. Although it proved to be effective in HIV-inhibition in vitro, it failed in cell-based assays due to low membrane permeability. To confer enhanced permeability, hydrocarbon stapling of CA1 was employed to form NYAD-1 [170,171]. These peptide inhibitors disrupt capsid particle assembly and, thus, the formation of immature and mature virus-like particles. They bind to a hydrophobic groove of the CA-CTD delimited by helices 8,9 and 11, as an amphipathic alpha helix, triggering an allosteric change in the dimer interface [170,172].

### 7.4. PF74

PF74 is a small organic compound that exhibits broad spectrum antiviral activity, discovered by Pfizer in 2010 by screening a chemical library for inhibitors of HIV-1 replication [138]. PF74 has a bimodal mechanism of inhibition that is concentration dependent [173]. At lower concentrations (<2 μM), it is suggested to directly compete with host cofactors needed for nuclear entry, in particular, Nup153 and CPSF6, as they share the same binding site [32,102]. This inference was made due to a reduction of the presence of 2-long-terminal-repeat (2-LTR) containing circles, which are markers of viral entry into the nucleus. At higher concentrations, PF74 disrupts replication at both early and late stages of the viral life cycle. It prevents reverse transcription from occurring but also disrupts mature capsid formation. It has been suggested that the latter is due to the premature destabilisation of the capsid core and the subsequent disruption caused to the NTD–CTD interface upon PF74 binding [173,174,175]. PF74 inserts into a novel binding site, specifically to a preformed pocket in the CA-NTD, which is delineated by the helices 3, 4, 5 and 7. However, recent studies also suggests that PF74 contacts the CA-CTD (NTD–CTD interface) of an adjacent CA subunit within the same hexamer [138,175]. Studies that examined the interaction of Nup153 with both monomeric CA and the CA hexamer revealed that Nup153 only has significant binding affinity to the hexamer [32]. From this, the inference can be made that disassembly of the CA hexamers also destroys the PF74 binding site, suggesting that the capsid docks with the nuclear pore in at least a partially intact, state. 

### 7.5. C-A1

Coumermycin A1 is a gyrase B inhibitor, developed by Roche in the 1970s, but identified as a potential HIV-1 inhibitor targeting CA in 2010 in a focused screen of known inhibitors, targeting ATP-dependent DNA motors. The bimodal mechanism by which C-A1 acts confers broad-spectrum inhibition across all HIV clades, as it impairs both HIV integration and gene expression. C-A1 also inhibits infection by binding to heat shock protein 90 (Hsp90). C-A1 shares the same binding pocket as PF74 and BI-1 on the CA-NTD, albeit an extended one. Further studies, using the Compute: Site Finder option in MOE, identified this binding groove on the capsid to include both capsid subunits and be delimited by Asn53, Asn57, Gln63, Lys70, Thr107 and Gln112 of subunit A and Glu128, Arg173, Gln179, Lys182 of subunit B [130]. C-A1 is reported to interfere with a post-entry step of the replication cycle but before integration [137].

### 7.6. Benzimidazoles: BD and BM/C1

The benzimidazole series compounds, BD and BM, were identified in 2012 in a screen for compounds that disrupted the in vitro assembly and association of CA-NC tubes on immobilised oligonucleotides. They both show a similar mechanism of action—BD compounds inhibit HIV-1 replication by blocking the assembly of immature virus particles and its subsequent release; BM compounds block the formation of mature capsid in released particles [176,177]. BD/BM shares the same deep, hydrophobic pocket as CAP-1 in the CA-NTD but triggers a further expansion of this pocket to facilitate binding. Optimisation of the BM series led to the discovery of compound-1 (C1). C1 binds in a shallow pocket near the base of the loop to which CypA binds [178,179,180].

### 7.7. BI-1 and BI-2

BI-1 and BI-2 are pyrrolopyrazolone compounds identified in a cell-based screen for post-entry inhibitors of single cycle HIV-1 infectivity [181]. The mechanism by which these compounds act has been putatively suggested to interfere with early post entry stages of viral replication following reverse transcription but prior to integration, suggesting that it interferes with the nuclear import of the PIC. BI-2 also shares its binding site with PF74 and C-A1, but since it is a smaller compound, it does not make contact with the adjacent CTD. Consequently, it could be hypothesised that, just like PF74, BI-2 also prevents the interaction of the HIV-1 core with host cofactors Nup153 and CPSF6 [32,182].

### 7.8. CK026/I-XW-053

CK026 was designed using a hybrid structure-based screening approach, which identified compounds that could inhibit HIV-1 replication by binding to the NTD–NTD intrahexamer interface. However, CK026 failed to inhibit HIV-1 replication in PBMCs, so a smaller analogue of this compound, I-XW-053, was developed, which proved to be more effective in PBMC-based assays [183]. I-XW-053 acts before integration, and PCR analysis revealed that treatment with I-XW-053 inhibited reverse transcription. Mutational analysis based on a docking model of I-XW-053 indicated that the novel binding site is located at the NTD-NTD interface [183,184].

### 7.9. Ebselen

Ebselen is an organoselenium compound identified in a screen for CA dimerization inhibitors. Ebselen binding results in impaired uncoating due to hyper stabilisation of the capsid and covalently binds to the CA-CTD. It is also suggested that Ebselen inhibits reverse transcription [185].

### 7.10. GS-CA1 and GS-6207

GS-CA1 was the predecessor to GS-6207, with the latter offering more potent inhibition of HIV-1 replication. Similar to PF74, they have a dose-dependent, multistage mechanism of antiviral action with broad spectrum inhibition across all HIV clades but with much greater potency. At low concentrations (50 pM), GS-6207 inhibits integration and, to a lesser extent, 2LTR circle formation. At a higher concentration (500 pM), GS-6207 inhibits release of viral particles. Both GS-CA1 and GS-6207 occupy the same binding site as PF74 and BI-2 at the NTD–CTD interface within CA hexamers. Since they also directly compete with Nup153 and CPSF6, nuclear ingress and integration are abrogated. Alternatively, GS-6207, by increasing the strength of the CA–CA interactions, may prevent proper disassembly of the capsid core and block integration. GS-6207 has high synergic effects and no cross-resistance with approved ARVs. Low systemic clearance in vivo and slow-release kinetics enable it to be administered via subcutaneous injection and orally. GS-6207 is the first compound that targets CA to progress into phase 2–3 of clinical trials [139].

## 8. Conclusions

Our understanding of the structure and function of HIV-1 CA has markedly advanced in the last few years. This has been made possible not only by the dedication and intelligence of many colleagues but also by technical breakthroughs, including super-resolution microscopy, live imaging, CryoEM, atomic force microscopy and next-generation sequencing. Often, it was the expert combination of these approaches that yielded the most spectacular and convincing results. Of course, there are several interesting questions in the field that one would like to address; for example, why have primate lentiviruses evolved to use CPSF6 for integration targeting? What is the functional significance of integration targeting, and is it relevant for the establishment of latency? What mechanisms are there to reduce sensing of the viral nucleic acids? How does an intact or partially disassembled capsid core go across the NPC selective barrier? How are mutant viruses such as N74D imported into the nucleus, and what actually happens if an “alternative” pathway is used? It has been an exciting time for all of us in the field, and it is hugely satisfying to see a highly promising new antiretroviral that targets CA reaching clinical trials. No less satisfying is seeing many junior investigators entering the field, an auspicious sign that many more insights are about to come.

## Figures and Tables

**Table 1:** Binding sites of selected compounds targeting HIV-1 capsid.

Compound	Binding Site	Structural Data 1	Structural Data 2
CAP-1 PDB: 2JPR [167]	CA-NTD: At the apex of a 5-helix bundle, in a deep hydrophobic cavity formed by the protuberance of the Phe32 side chain	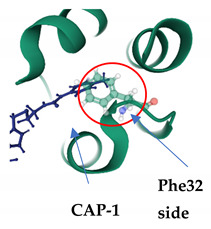	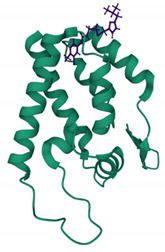
NYAD-1 PDB: 2L6E [170]	CA-CTD	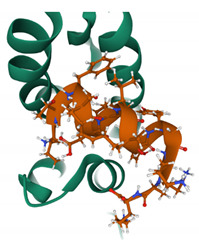	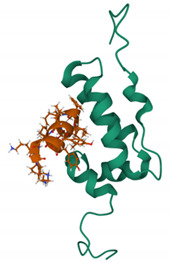
PF74 PDB: 4XRQ [186]	NTD–CTD interface	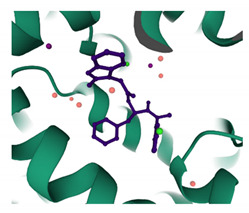	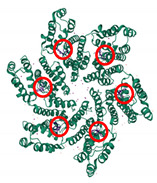
GS-6207 PDB: 6VKV [187]	NTD–CTD interface	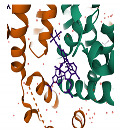	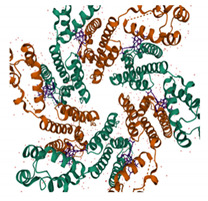

## Data Availability

Not applicable.
